# SOCIAL ISOLATION, UNMET NEEDS, AND LONG-TERM SERVICES AND SUPPORTS IN COMMUNITY-DWELLING OLDER ADULTS

**DOI:** 10.1093/geroni/igad104.2040

**Published:** 2023-12-21

**Authors:** Mary Louise Pomeroy, Andrew Jopson, Jenni Reiff, M J Wu, Katherine Ornstein, Thomas Cudjoe, Chanee Fabius

**Affiliations:** Johns Hopkins University, Baltimore, Maryland, United States; Johns Hopkins University, Baltimore, Maryland, United States; Johns Hopkins University, Baltimore, Maryland, United States; Johns Hopkins University, Baltimore, Maryland, United States; Johns Hopkins University, Baltimore, Maryland, United States; Johns Hopkins University School of Medicine, Baltimore, Maryland, United States; Johns Hopkins University, Baltimore, Maryland, United States

## Abstract

Social isolation is associated with adverse health outcomes among older adults but can be attenuated through increased social contact and support. Long-term services and supports (LTSS) are a potential mechanism to increase social contact and provide socially isolated older adults with the help needed to perform routine activities. Using a nationally representative sample of 6,705 community-dwelling older adults from the 2015 National Health and Aging Trends Study (NHATS), we examined associations between social isolation, functional difficulty, adverse consequences due to unmet needs, and indicators of the LTSS environment (e.g., Meals on Wheels). About 22% of older adults were socially isolated. Socially isolated older adults had greater functional difficulty compared to those who were not socially isolated (45.3% vs 38.3%; p<0.0001). Socially isolated older adults were more likely to be Medicaid enrollees (17.5% vs 8.4%; p<0.0001) and use LTSS including SNAP benefits (12.7% vs 5.5%; p<0.0001), Meals on Wheels (4.1% vs 1.6%; p<0.0001), energy/gas financial assistance (7.5% vs 4.2%; p<0.0001), and transportation services for older adults or people with disabilities (4.6% vs 2.7%; p=0.001). Despite greater LTSS use, socially isolated older adults more often experienced adverse consequences due to unmet needs compared to those who were not socially isolated (12.5% vs 7.7%; p<0.0001). Findings suggest that LTSS in the form of social and senior services are critical in supporting those lacking social connection, but they may not be adequate to support aging in place in the absence of informal support.

